# The comparative importance of mental and physical disorders for health-related days out of role in the general population of Saudi Arabia

**DOI:** 10.1186/s12889-022-12721-z

**Published:** 2022-02-12

**Authors:** Yasmin A. Altwaijri, Victor Puac-Polanco, Abdullah S. Al-Subaie, Yasser Ad-Dab’bagh, Abdul Hameed Al-Habeeb, Lisa Bilal, Nancy A. Sampson, Andrew J. King, Somnath Chatterji, Ronald C. Kessler

**Affiliations:** 1 Biostatistics, Epidemiology and Scientific Computing Department, King Faisal Specialist Hospital and Research Centre, Riyadh, Saudi Arabia; 2 King Salman Center for Disability Research, Riyadh, Saudi Arabia; 3 SABIC Psychological Health Research & Applications Chair (SPHRAC), College of Medicine, King Saud University, Riyadh, Saudi Arabia; 4 Department of Health Care Policy, Harvard Medical School, 180 Longwood Avenue, Boston, MA 02115 USA; 5 Edrak Medical Center, Riyadh, Saudi Arabia; 6 Mental Health Department, King Fahad Specialist Hospital-Dammam, Dammam, Saudi Arabia; 7 Vision Realization Office, Ministry of Health, Riyadh, Saudi Arabia; 8 National Center for Mental Health Promotion, Ministry of Health, Riyadh, Saudi Arabia; 9 Department of Information, Evidence, and Research, World Health Organization, Geneva, Switzerland

**Keywords:** Saudi Arabia, Mental disorder, Physical disorders, Days out of role, Impairment, Saudi National Mental Health Survey (SNMHS)

## Abstract

**Background:**

A major component of illness burden is role impairment. As part of the recently-completed Saudi National Mental Health Survey (SNMHS), we compare the number of days out of role in the Saudi population associated with ten core mental disorders assessed in the survey to those associated with ten commonly occurring chronic physical disorders.

**Methods:**

The SNMHS was a household survey that assessed prevalence of ten common anxiety, mood, disruptive behavior, and eating disorders in a nationally representative sample of *n* = 1981 citizens of the Kingdom of Saudi Arabia (KSA) ages 15–65. Comparison information was obtained on prevalence of ten common chronic physical disorders and number of health-related days out of role (DOR) in the 30 days before interview. Generalized linear models were used to examine univariate and multivariable associations of disorders with DOR and to calculate population attributable risk (PAR) separately and overall for the disorders controlling for socio-demographics.

**Results:**

19.9% of respondents had one or more of the selected mental disorders and 47.1% had one or more of the selected physical disorders. Nine mental disorders and two physical disorders were associated with increased DOR. PAR was 32.9% for mental disorders, 27.0% for physical disorders, and 59.9% for both combined.

**Conclusions:**

Mental disorders are associated with a substantial proportion of all health-related DOR in the Kingdom of Saudi Arabia. Programs to detect and treat mental disorders might lead to substantially decreased role impairment in the Kingdom.

**Supplementary Information:**

The online version contains supplementary material available at 10.1186/s12889-022-12721-z.

## Background

Days out of role (DOR) are an important component of illness burden [1–4]. While only small variations have been found in the proportion of individuals reporting health-related DOR across high-income Western countries [5], estimates specific to individual countries are necessary to make accurate health policy decisions about the cost-effectiveness of various initiatives to reduce disease burden. The Kingdom of Saudi Arabia (KSA) has committed to developing a new healthcare system that meets the social, mental, and physical needs of its population [6]. Estimates from the Global Burden of Disease (GBD) Study suggest that mental disorders need to be a central component in these plans given that this class of disorders is estimated to play a key role in the disease burden of the KSA population [7, 8]. However, GBD estimates were based on extrapolations from data on mental disorders collected in other Arab countries rather than on data from KSA. The Saudi National Mental Health Survey (SNMHS) was carried out to address this limitation by producing direct estimates of mental disorder prevalence and burden. Initial SNMHS reports documented high prevalence of common mental disorders [9, 10] and low rates of treatment [11, 12]. The current report presents the first data on the societal burden of these disorders.

DOR is recognized as a major indirect cost of illness due to its high economic impact on individuals, employers, and society [13]. Measures of DOR are also convenient in that they can be reported with good accuracy over short recall periods (e.g., the 30 days before an interview) [14] and provide a standardized metric that can be compared across different disorders and contexts [5]. Moreover, studies of DOR based on nationally representative samples allow for reliable estimations that account for variations between disorders produced by severity, seeking treatment behaviors, and comorbidity.

Existing evidence is limited regarding impairments in role functioning associated with mental and physical disorders in the Eastern Mediterranean region. We are aware of only one such study, which was carried out in Iraq [2]. That study assessed the societal burden of common mental and chronic physical disorders in a general-population survey and found that bipolar disorder, substance use disorder, neurological disorders (multiple sclerosis, Parkinson’s, and epilepsy or seizures), and cancer were associated with the highest individual-level numbers of DOR. Extrapolating these findings to KSA would be inappropriate, though, given the enormous differences between the two countries [15]. Data also exist for Lebanon that have been reported only pooled with other countries as part of a cross-national analysis [5]. But Lebanon, like Iraq, would not be an appropriate comparator for KSA given that both Lebanon and Iraq are middle-income countries [16], whereas KSA is one of the top 20 economies in the world [17] and the largest economy in the Arab World [18]. This is the first study to report on the associations of mental and physical disorders with days out of role using large-scale general-population data in the KSA.

## Methods

### Sample

The SNMHS is a mental health needs assessment survey carried out in a nationally representative sample of households in KSA. The survey focuses on citizens (i.e., excluding guest workers and their families) aged 15–65 living in the KSA between 2011 and 2016 [19]. The SNMHS was implemented as one surveys in the World Health Organization (WHO) World Mental Health (WMH) Survey Initiative [20]. Fieldwork was conducted 2014–2016, with pauses for Ramadan and the summer (due to very high temperatures), The sample included 4302 households in 11 of the 13 administrative areas of the country. The other two administrative areas (Jazan & Najran) were excluded because of a political conflict at the time of the survey.

The survey administered interviews face-to-face to respondents in their homes. Like other WMH surveys, the SNMHS used a two-part design within the interview to reduce burden among respondents who did not have mental disorders. Part I, which assessed core disorders, was administered to all *n* = 4004 respondents. Part II, which assessed disorders of secondary interest and a wide range of correlates, was then administered to all Part I respondents who met lifetime criteria for any of these disorders plus a probability sub-sample of other Part I respondents (*n* = 1981). Importantly, a weight was used for Part II respondents who did not have a core Part I disorder equal to the inverse of their probability of selection into Part II. This adjusted for the under-sampling of these non-cases and restored the same prevalence estimates in the weighted Part II sample as in the Part I sample. This weighted Part II sample is used in the current report.

The SNMHS sample design [6] and field procedures [21] have been reported in detail elsewhere. In brief, though, the survey was based on a stratified multistage clustered area probability design in which Primary Sampling Units (PSUs) were constructed separately within each of the 11 administrative areas based on census counts based on maps provided and updated by the Ministry of Economy and Planning [22]. After establishing a minimum number of PSUs to be selected in each of the smaller strata, the rest of the PSUs were allocated approximately proportionate to size of the Saudi household population across administrative areas according to the 2010 Census [19]. Households were then selected using systematic selection from an address-based sampling frame within PSUs. At the household level, one respondent of each sex was selected at random from all those eligible (i.e., Saudi citizen, ages 15–65, able to speak Arabic). A within-household probability of selection weight was assigned to each respondent to adjust for variation in probability of selection depending on number of eligible people of the same sex in the household. The response rate (RR) was 61% based on the American Association of Public Opinion Research RR2 definition [23]. This is comparable to the WMH survey RRs in other high-income countries [24].

### Measures

#### Mode of administration

The Saudi adaptation of the computerized Composite International Diagnostic Interview 3.0 (CIDI [25];) was used to administer the interviews by trained lay interviewers. The CIDI is a fully structured diagnostic interview documented to yield valid diagnoses of common mental disorders [26]. Given the length and complexity of the interview, a computer-assisted personal interview (CAPI) mode was used for administration [27]. In addition, an audio computer-assisted self-interviewing (ACASI) method was used for sensitive sections, such as those asking about suicidality, based on evidence that this approach leads to increased reports of sensitive information [28].

The mental disorders assessed in the CIDI were based on both ICD-10 [29] and DSM-IV [30] diagnostic criteria, only DSM-IV criteria are used here. We focus on 12-month prevalence of five anxiety disorders (panic disorder or agoraphobia, separation anxiety or social phobia, generalized anxiety disorder, post-traumatic stress disorder, obsessive-compulsive disorder), two mood disorders (major depressive disorder, bipolar I-II disorder), two disruptive behavior disorders (attention-deficit/hyperactivity disorder (ADHD), intermittent explosive disorder), and any eating disorder (anorexia nervosa, binge eating disorder, bulimia nervosa). A clinical reappraisal study showed that CIDI diagnoses are valid but conservative compared to diagnoses based on blinded clinical reappraisal interviews with the Structured Clinical Interview for DSM-IV [31].

#### Physical disorders

Common physical disorders were classified according to a chronic disorders checklist adapted from a standard list [32]. Such checklists have been found to be more accurate than open-ended questions in assessing prevalence of physical conditions [33, 34]. We focused on 12-month prevalence and assessed ten chronic physical disorders known to be commonly occurring and seriously impairing: arthritis, cancer, cardiovascular disorders (heart disease, hypertension), chronic pain conditions (back or neck pain, other chronic pain), headaches, respiratory disorders (asthma, other lung diseases), and injuries requiring hospitalization.

#### Days out of role

A modified version of the WHO Disability Assessment Schedule [35, 36] was used to ask about number of days in the 30 days before the interview (i.e., beginning yesterday and going back 30 days) when respondents were totally unable to work or carry out their normal activities because of problems with either their mental or physical health. Prior research has documented good concordance of such self-reports with payroll records of employed people [37, 38] and prospective daily diary reports [39]. Partial days out of role were also assessed over the same recall period as days when respondents cut back on the quality of their work or how carefully they worked because of problems with either their mental or physical health. Total days out of role were defined as the sum of number of full days plus 0.5 of number of partial days out of role.

#### Socio-demographics

The socio-demographic correlates considered included age, gender, education, employment, and marital status. Age was coded in the four categories 15–24, 25–34, 35–49, and 50–65 years of age. Gender was coded female and male. Education distinguished respondents who were students at the time of interview and non-students and categorized them into low (0–6 years of education), low-average (7–9 years of education), high-average (10–15 years of education), and high education (16+ years of education). Note that the youngest respondents who were still students were classified as having high-average education even though some of them will go on to higher education. This means that the education measure assesses education to date. Employment was a dichotomous variable for currently working yes or no, again noting that students were coded as not working. Marital status was coded as either never married, married, or previously married (either separated, divorced, or widowed).

### Statistical analysis

Generalized linear models (GLMs) were used to estimate the associations of dummy variables for disorders with the summary DOR measure controlling for socio-demographic factors. To account for the skewness of the outcome variable, which was coded in the range 0–30, we evaluated seven different combinations of the link function (linear, logarithmic, square root) and error structures (constant, error variance proportional to the mean, error variance proportional to the mean squared). Standard diagnostic procedures for model comparisons [40] showed that ordinary least squares (OLS) model fit statistics were almost identical to other more complex GLMs. OLS was consequently used in the analysis. We adjusted for the geographic clustering and weighting of the data by using the design-based Taylor series linearization method [41].

We used four models to estimate the associations of disorders with DOR. (i) Univariable models examined associations of each disorder with DOR controlling for age, gender, education, employment, and marital status but without including more than one disorder in the model. (ii) A multivariable model examined additive associations of all disorders together. (iii) High comorbidity among disorders, which has also been found in prior research worldwide [42], led to instability in individual coefficients in the additive multivariable model. We addressed this by estimating a lasso penalized regression model [43] for selecting a more parsimonious set of predictors. (iv) A nonadditive multivariable model evaluated the possibility of significant interactions among the disorders. Given that the number of possible combinations of comorbid disorders (2^20^–20 = 1,048,556) far exceeded the number of respondents, it was necessary to impose some structure on the comorbidity parameters in this model. We did this using the random forest (RF) [44, 45] machine-learning algorithm to search for stable interactions among disorders in predicting DOR. The cross-validated predicted outcome score based on RF was then added to the additive multivariable model to evaluate the overall importance of interactions. SAS software Version 9.4 [46] was used for the GLMs and R Version 3.6.3 [47] was used for the lasso and random forest models.

Population attributable risk percent (PARP) was calculated for all 20 disorders combined and separately for the ten mental and ten physical disorders [48]. Incremental annualized per person total disability days were also estimated by multiplying PARP by mean DOR and projecting from 30 days to 12 months separately for the full sample and respondents with individual disorders. A SAS macro was used to calculate PARPs and their standard errors. These standard errors were estimated using the jackknife repeated replications (JRR) simulation method [49].

Statistical significance was evaluated using .05 level two-sided. No corrections were made for multiple comparisons.

## Results

### Sample characteristics

The socio-demographic distribution of the sample (Table 1) was comparable to KSA Census population data in terms of a younger age distribution than in most other developed countries, high education, and a very high rate of not working. The high number not working is likely due to a combination of the young age of the sample, presence of many students, a low proportion of females in the workforce, a low proportion of Saudi nationals in low wage jobs dominated by expatriates, and an unemployment rate above 10%.

**Table 1:** Respondent characteristics in the Part II sample of the Saudi National Mental Health Survey (*n* = 1981)^a^

	Distribution	
	%	(SE)
**Age**		
15–24	35.5	(2.1)
25–34	23.8	(1.8)
35–49	27.7	(2.0)
50+	13.0	(1.3)
**Gender**		
Male	50.5	(1.4)
Female	49.5	(1.4)
**Employment**		
Working	38.5	(1.8)
Not working/other	61.5	(1.8)
**Education**		
Student	12.8	(1.3)
Low	16.1	(1.7)
Low-average	13.2	(1.4)
High-average	38.2	(2.0)
High	19.7	(2.6)
**Marital status**		
Previously	10.9	(1.2)
Never	43.5	(2.0)
Currently	45.5	(2.0)

*Abbreviations*: *SE* standard error

^a^Estimates based on weighted Part II data

### The distribution of days out of role

The mean number of total DOR (i.e., combining full and partial DOR) was 1.3 in the 30 days before the interview. (Table 2) 19.2% of all respondents reported any DOR, with a mean of 7.0 such days among those reporting any. The median (2.0) was lower than the mean, indicating a right-skewed distribution.

**Table 2:** Distribution of total days out of role (DOR) in the Part II Saudi National Mental Health Survey (n = 1981)^a^

	Distribution of DOR	
	Est	(SE)
**Days out of role (%)**^**b**^		
Any	19.2	(1.4)
0.5–1.5	5.0	(0.7)
2.0–2.5	3.2	(0.5)
3–5	4.6	(0.6)
6–10	3.0	(0.4)
11–20	1.5	(0.2)
21–30	2.0	(0.6)
**Total**		
Mean^c^	1.3	(4.6)
Mean/Any^d^	7.0	(7.0)
Median^c^	2.0	–

*Abbreviations*: *SE* standard error

^a^Estimates based on weighted Part II data. Total DOR are defined as the sum of (1) full days and (2) partial days divided by 2

^b^Total estimates that included non-integer values (i.e., values = 2.5) based on partial DOR were rounded down to the nearest integer in reporting distributions. This downward rounding was not applied to the calculations of mean and median

^c^Among all Part II sample (*n* = 1981)

^d^Among Part II respondents with any DOR: full days (*n* = 366); partial days (*n* = 470); either (*n* = 544)

### Twelve-month prevalence of disorders

An estimated 19.9% of respondents met DSM-IV criteria for any of the mental disorders considered here, whereas 47.1% met criteria for at least one of the physical disorders and 55.5% for at least one of either type of disorder. (Table 3) Separation anxiety/social phobia was the most prevalent mental disorder (7.5%), followed by major depressive disorder (3.8%), ADHD (3.5%), and eating disorder (3.2%). Chronic headache was the most prevalent chronic physical disorder (29.0%), followed by back/neck pain (11.4%), arthritis (8.9%), and other chronic pain conditions (8.4%). The number of respondents reporting other lung disease and cancer (*n* = 6 each) is too small for stable analysis of disorder-specific associations.

**Table 3:** Twelve-month prevalence and monthly estimated total days out of role (DOR) among respondents with each of the mental and physical disorders in the Part II Saudi National Mental Health Survey (n = 1981)^a^

	Prevalence		Total DOR		
	%	(SE)	Mean	SD	(n)
**I. 12-month mental disorders**					
A. Anxiety disorder					
Panic disorder or Agoraphobia	2.8	(0.4)	3.4	5.3	(106)
Separation anxiety or Social phobia	7.5	(0.7)	3.3	5.5	(205)
Generalized anxiety disorder	0.9	(0.2)	4.2	6.0	(32)
Post-traumatic stress disorder	1.8	(0.3)	3.6	4.4	(61)
Obsessive-compulsive disorder	1.8	(0.3)	3.6	5.1	(64)
Any anxiety	12.3	(1.0)	3.3	5.4	(367)
B. Mood disorder					
Major depressive disorder	3.8	(0.4)	4.7	5.3	(158)
Bipolar I-II disorders	3.0	(0.5)	3.6	5.5	(85)
Any mood	6.8	(0.7)	4.2	5.4	(243)
C. Disruptive behavior disorder					
ADHD	3.5	(0.7)	4.2	4.9	(112)
Intermittent explosive disorder	2.7	(0.6)	4.9	6.5	(72)
Any disruptive behavior	5.6	(0.8)	2.7	4.7	(168)
D. Any eating disorder	3.2	(0.5)	3.5	6.6	(91)
E. Any mental^b^	19.9	(1.3)	3.3	5.3	(622)
**II. 12-month physical disorders**					
Arthritis	8.9	(1.4)	3.9	7.4	(222)
Chronic back/neck pain	11.4	(1.0)	2.2	4.3	(311)
Chronic headaches	29.0	(2.0)	1.6	4.1	(719)
Other chronic pain	8.4	(0.9)	2.4	4.1	(241)
Hypertension	6.8	(0.9)	1.8	4.0	(164)
Heart disease	1.5	(0.6)	12.4	13.1	(34)
Asthma	4.5	(0.6)	2.4	5.2	(117)
Other lung disease	0.6	(0.5)	28.9	7.3	(6)
Injuries requiring hospitalization	6.4	(0.9)	2.5	5.5	(172)
Cancer	0.5	(0.4)	1.1	4.7	(6)
Any physical	47.1	(2.2)	2.0	5.0	(1136)
**III. Total**					
Only mental	7.4	(0.7)	3.4	6.3	(194)
Only physical	34.6	(1.9)	1.5	5.1	(708)
Both mental and physical	12.5	(2.5)	3.3	4.8	(428)
No mental or physical	45.5	(2.5)	0.4	2.2	(651)
Total	–	–	1.3	4.6	(1981)

*Abbreviations*: *SE* standard error, *SD* standard deviation, *ADHD* attention-deficit/hyperactivity disorder

^a^Estimates based on weighted Part II sample data. Total DOR are defined as the sum of (1) full days and (2) partial days divided by 2.

^b^The prevalence estimate is lower than in previous SNMHS reports (Al-Habeeb et al., 2020; Altwaijri et al., 2020a) because we focus here only on the 10 most common mental disorders in the SNMHS.

### Days out of role associated with the disorders

The mean number of total DOR was 0.4 among respondents with none of the disorders considered here, 3.4 among those only with mental disorders, 1.5 among those only with physical disorders, and 3.3 among those with both mental and physical disorders. (Table 3) The mean number of DOR among respondents with each of the ten mental disorders was in the 3.3–4.9 range. Means for seven of the ten physical disorders were lower (1.1–2.5), with means of 3.9 for arthritis, 12.4 for heart disease, and 28.9 for other (than asthma) lung disease, but again noting that the number of respondents with other lung diseases was too small for stable analysis of the disorder-specific association with DOR.

### Regression models for the associations of disorders with total days out of role

Nine of the ten univariate regression coefficients of mental disorders with total DOR adjusting for socio-demographics were statistically significant (b = 2.18–3.92). The exception was a non-significantly elevated coefficient for eating disorders (b = 2.48). (Table 4) Only two of these associations remained significant, though, in the multivariable additive model that included all ten mental disorders as predictors: b = 2.78 for major depressive disorder; and 2.00 for ADHD. Two of the ten univariable coefficients for physical disorders were significant (b = 1.01 for other [than a series of explicit pain conditions] chronic pain; b = 27.80 for other [than asthma] lung disease). Both remained significant in the multivariable additive model (b = 0.75 for other chronic pain; b = 26.99 for other lung disease). The lasso penalized regression model (Supplementary Table 1) verified the unique importance of these four conditions for total DOR. The random forest model found no evidence of significant interactions among disorders predicting DOR (Supplementary Table 1), although this might have more to do with the low power to detect such associations with a sample of the size we have here than with the absence of interactions in the population. We also examined the separate associations of disorders with full and partial DOR, but no disorders emerged as significant that were not seen in the models for total DOR (Supplementary Tables 2–3).

**Table 4:** Incremental increases in total days out of role (DOR) associated with each of the mental and physical disorders in the Part II Saudi National Mental Health Survey (n = 1981)^a^

	Univariable model			Multivariable model		
	Coefficient	(95% CI)	c^**2**^	Coefficient	(95% CI)	c^**2**^
**12-month mental disorders**						
Anxiety disorder						
Panic disorder or Agoraphobia	2.18	(0.11–4.25)	4.3*	0.86	(−1.19–2.92)	0.7
Separation anxiety or Social phobia	2.28	(1.08–3.48)	14.0*	1.18	(−0.15–2.51)	3.1
Generalized anxiety disorder	2.82	(0.01–5.62)	3.9*	1.35	(−1.95–4.66)	0.7
Post-traumatic stress disorder	2.29	(0.40–4.19)	5.7*	0.98	(−0.85–2.82)	1.1
Obsessive-compulsive disorder	2.43	(0.15–4.72)	4.4*	0.99	(−1.52–3.50)	0.6
Mood disorder						
Major depressive disorder	3.61	(1.38–5.85)	10.2*	2.78	(0.50–5.06)	5.8*
Bipolar I-II disorders	2.56	(0.52–4.61)	6.1*	0.54	(−1.48–2.56)	0.3
Disruptive behavior disorder						
ADHD	3.24	(1.27–5.21)	10.5*	2.00	(0.23–3.77)	5.0*
Intermittent explosive disorder	3.92	(1.54–6.31)	10.6*	1.89	(−0·62–4.39)	2.2
Any eating disorder	2.48	(−0.54–5.49)	2.6	1.28	(−1.61–4.17)	0.8
**12-month physical disorders**						
Arthritis	2.42	(−0.27–5.11)	3.2	0.37	(−0.40–1.14)	0.9
Back/neck pain	0.74	(−0.20–1.67)	2.4	0.15	(−0.57–0.87)	0.2
Headaches	0.36	(−0.27–0.99)	1.3	−0.02	(− 0.53–0.48)	0.0
Other chronic pain	1.01	(0.19–1.82)	5.9*	0.75	(0.13–1.37)	5.6*
Hypertension	−0.19	(−1.59–1.21)	0.1	− 0.04	(− 0.71–0.63)	0.0
Heart disease	10.78	(−2.04–23.60)	2.8	0.80	(−1.06–2.66)	0.7
Asthma	0.98	(−0.71–2.67)	1.3	0.24	(−1.36–1.83)	0.1
Other lung disease	27.80	(25.25–30.35)	463.5*	26.99	(23.10–30.88)	188.1*
Injuries requiring hospitalization	1.46	(−0.33–3.26)	2.6	1.20	(−0.68–3.08)	1.6
Cancer	0.02	(−1.99–2.04)	0.0	0.30	(−1.32–1.93)	0.1

*Abbreviations*: *CI* confidence interval, *ADHD* attention-deficit/hyperactivity disorder

*Significant at the .05 level, two-sided test

^a^Estimates come from linear regression models based on weighted Part II sample data. Total DOR are defined as the sum of (1) full days and (2) partial days divided by 2. All models included controls for age, gender, employment, education, and marital status. The 20 univariable models added only one mental or physical disorder as a predictor to these control variables. The multivariable model added all 20 disorders to the control variables

### Population attributable risk proportions

Population attributable risk proportions (PARP) estimated that roughly one-third (32.9%) of the total DOR in the KSA are associated with the ten mental disorders considered here controlling for the ten physical disorders, 27% of DOR are associated with the ten physical disorders controlling for the ten mental disorders, and 59.9% of DOR are associated with one or more of the 20 disorders. (Table 5) The remaining 40.1% of DOR are presumably due to the many other chronic and acute ill health conditions not assessed in our short battery. Mean incremental annualized per person estimates suggest that the eradication of all the mental disorders considered here would result in a decrease of 5.3 annual DOR per capita due to a decrease of 26.7 DOR among the 19.9% of the population with these mental disorders. The same calculations suggest that eradication of all the physical disorders considered here would result in a decrease of 4.4 annual DOR per capita due to a decrease of 9.3 such days among the 47.1% of the population with these disorders. Results were roughly comparable when we repeated the PARP analysis separately for full DOR and partial DOR and to those from total DOR (Supplementary Table 4).

**Table 5:** Population attributable risk proportion (PARP) and incremental annualized per person total days out of role (DOR) associated with the mental and physical disorders in the Part II Saudi National Mental Health Survey (n = 1981)^a^

	12-month mental disorders		12-month physical disorders		Neither	
	Estimate	(SE)	Estimate	(SE)	Estimate	(SE)
**I. Prevalence (%)**						
Total days out of role	19.9	(1.3)	47.1	(2.2)	45.5	(2.5)
**II. PARP (%)**^**b**^						
Total days out of role	32.9	(6.6)	27.0	(8.5)	40.1	(5.7)
**III. Inc**re**mental annualized per person total disability days (total sample)**^**c**^						
Total days out of role	5.3	(1.1)	4.4	(1.4)	–	–
**IV. Incremental annualized per person total disability days (among people with the disorders)**^**d**^						
Total days out of role	26.7	(5.3)	9.3	(2.9)	6.5	(0.9)

^a^Estimates are based on weighted Part II sample data. Total DOR are defined as the sum of (1) full days and (2) partial days divided by 2

^b^PARP estimates are generated from the multivariable model in Table 4

^c^The estimates of annualized days are based on PARP estimates multiplied by the mean total DOR in the last 30 days times 12. As reported in Table 1, this mean for the past 30 days is 1.3

^d^Sub-group annualized days equal total annualized days multiplied by 1/(prevalence of the subgroup in the total sample)

## Discussion

The results reported here are the first to examine the associations of mental and physical disorders with DOR in the KSA. Only one prior study attempted to estimate role impairments due to mental and physical disorders in the KSA. That study, carried out by the Global Burden of Diseases (GBD) collaborators, focused on people ages 15–44 based on indirect estimates from meta-analyses of diverse data across all countries in the eastern Mediterranean region [8]. The report focused on the ten leading causes of disability-adjusted life years (DALYs) and estimated that major depressive disorder was the leading cause of DALYs (7.7%) among females. Major depressive disorder was estimated in the GBD report to be the sixth leading cause of DALYs in KSA between 1990 and 2010 among males. Anxiety disorders were also included as a leading cause of disability, in the ninth position, attributed with 2.9% of total DALYS for both genders in 2010. However, the analysis made no attempt to examine other mental disorders or make comparisons between the different causes of disease and disability measures.

The lack of disability data derived from accurate local epidemiological studies of mental disorders and the lack of specific DOR data related to common mental disorders has contributed to the perceived relative lack of parity in the planning of services and allocation of investments for the sector of healthcare focused on mental health as well as disability supports provided in KSA. Our results provide much more fine-grained data arguing for the importance of mental disorders for total DOR in the KSA based on more direct measures and a range of statistical models to check modeling assumptions. We estimate that more than half (PARP = 59.9%) of DOR in the population are associated with the 20 disorders included in our analysis. Despite the lower prevalence of mental disorders (19.9%) compared to physical disorders (47.1%), mental illnesses are associated with a higher proportion of DOR (32.9%) than the physical disorders considered here (27.0%). This finding differs from surveys in other high-income countries using comparable measures, which typically find that physical disorders account for a higher proportion of DOR than do mental disorders [5]. This difference presumably reflects the very distinctive early age structure of KSA compared to the great majority of other high-income countries, where a much higher proportion of the population are elderly. Chronic physical disorders of the sort considered here increase in prevalence relative to mental disorders with increasing age in all countries, accounting for the greater relative importance of mental than physical disorders in KSA than other high-income countries.

These results confirm other evidence that mental disorders are an important source of role impairment in the KSA [8] and suggest that it could be important for the currently planned and ongoing reforms of the Saudi healthcare system to address this class of disorders. At the time of preparation of this manuscript, a National Strategy for Mental Health, Addictions and Developmental Disorders has been proposed to reform the relevant services in KSA over a period of 20 years. The implementation of this strategy within the healthcare sector delivered by the Ministry of Health has already begun to be implemented [50]. Data derived from the SNMHS were instrumental in justifying many of the planned components of this evolving strategy. Given the macro-economic impact of the loss of nearly one-third of human resource capital being directly related to the mental disorders examined in this study, we expect the present analysis to significantly bolster the case for a substantial investment and increased emphasis on the prevention, early detection, early intervention, rehabilitation, and overall management of the 10 mental disorders included in it. In addition, we expect the present analysis to potentially influence the policies and the allocation of resources and supports by the agencies providing social service, vocational rehabilitation and training, and unemployment benefits.

Our results must be interpreted within the context of important study limitations. First, prevalence estimates of mental disorders were lower when compared to other high-income countries [5]. This is true despite the earlier age structure of KSA, which, as noted above, typically is associated with increased prevalence of mental disorders. One possible reason for this is that stigma, which is high for mental disorders in KSA, could have led to underreporting [51, 52]. If so, our estimates of the relative burden of mental disorders on DOR are likely conservative. Another possibility is that the diagnostic instrument used (CIDI 3.0), which is known to produce conservative estimates more generally, might have been even more conservative in KSA [31]. It is noteworthy, though, that underreporting of physical disorders might have occurred as well due to the use of self-report physical disorder checklists rather than medical examinations, but we would expect this to be less of a problem than for reports of mental disorders because of the lower stigma associated with physical than mental disorders and evidence from previous research that good correspondence exists between self-reports and clinical records of chronic physical disorders [53, 54].

Second, we only included ten mental and ten physical disorders in our analysis, although our PARP estimates show that these disorders account for almost 60% of all health-related DOR in the population. Even if all of the remaining 40% of DOR are due to physical disorders, the 32.9% associated with mental disorders represents an important source of lost human capital in the Saudi population. Third, biases from self-reports about DOR are possible, although the short recall period and the clear categorical description of full DOR (i.e., “totally unable to work or carry out your normal activities”) would be expected to reduce any such bias. Fourth, the focus on DOR might have obscured less severe impairments in role functioning that, if assessed, might have painted a different portrait of the relative importance of mental and physical disorders. Future research is needed to investigate this possibility. Fifth, although we implicitly interpreted the associations of disorders with DOR as causal, the analysis was based on an observational cross-sectional sample.

## Conclusions

Within the context of these limitations, the present study is the first to report on role impairment associated with mental and physical disorders in the KSA. We found consistent associations between mental disorders and days out or role. Despite the definitive evidence of these associations being causal, these results, when coupled with knowledge that treatment rates of people with mental disorders in the KSA are low [12], highlights the need for programs designed to prevent and treat mental illnesses in the Kingdom. It would be valuable for the KSA’s new healthcare system to launch a health promotion campaign to increase awareness among the general public about the serious impact of mental disorders on day-to-day role functioning and the need to seek early treatment in conjunction with the new initiatives noted above. Given the ongoing reforms in mental health services, an evaluation of the extent to which the introduced reforms lead to reductions in the burden of mental disorders measured by various indices of disability or lost productivity, including DOR, could then be carried out. It would also be useful to evaluate the extent to which the interventions reduce prevalence of physical disorders, allowing uncertainties to be addressed about the role of mental disorders as causal risk factors for mental-physical comorbidity.

## Supplementary Information

**Additional file 1: Supplementary Table 1.** Incremental increases in total days out of role (DOR) associated with each of the mental and physical disorders in the Part II Saudi National Mental Health Survey (*n* = 1981)^a^. **Supplementary Table 2.** Incremental increases in full days out of role (DOR) associated with each of the mental and physical disorders in the Part II Saudi National Mental Health Survey (*n* = 1981)^a^. **Supplementary Table 3.** Incremental increases in partial days out of role (DOR) associated with each of the mental and physical disorders in the Part II Saudi National Mental Health Survey (n = 1981)^a^. **Supplementary Table 4.** Population attributable risk percent (PARP) and incremental annualized per person full and partial days out of role (DOR) associated with the mental and physical disorders in the Part II Saudi National Mental Health Survey (n = 1981)^a^.

## Data Availability

A public use dataset is not available because of restrictions in the informed consent language used to recruit respondents and WMH consortium agreements. However, a de-identified minimal dataset for quality assurance can be obtained by contacting the first author at yasmint@kfshrc.edu.sa.

## Abbreviations

SNMHS: Saudi National Mental Health Survey
KSA: Kingdom of Saudi Arabia
DOR: health-related Days Out of Role
PAR: Population Attributable Risk
GBD: Global Burden of Disease
RR: Response Rate
WHO: World Health Organization
WMH: World Mental Health
DSM-IV: Diagnostic and Statistical Manual of Mental Disorders, fourth edition
ADHD: Attention-deficit/Hyperactivity disorder
CIDI: WHO Composite International Diagnostic Interview
GLM: Generalized linear models
OLS: Ordinary Least Squares
PARP: Population Attributable Risk Percent
JRR: Jackknife Repeated Replications
DALYs: Disability-Adjusted Life Years
