# Supplementary material for: The comparative importance of mental and physical disorders for health-related days out of role in the general population of Saudi Arabia
Source: BMC Public Health. 2022 Feb 12;22:289. doi: 10.1186/s12889-022-12721-z (PMC8840674; doi:10.1186/s12889-022-12721-z)
Supplement: Supplementary file 1 — Additional file 1: Supplementary Table 1. Incremental increases in total days out of role (DOR) associated with each of the mental and physical disorders in the Part II Saudi National Mental Health Survey (n = 1981)a. Supplementary Table 2. Incremental increases in full days out of role (DOR) associated with each of the mental and physical disorders in the Part II Saudi National Mental Health Survey (n = 1981)a. Supplementary Table 3. Incremental increases in partial days out of role (DOR) associated with each of the mental and physical disorders in the Part II Saudi National Mental Health Survey (n = 1981)a. Supplementary Table 4. Population attributable risk percent (PARP) and incremental annualized per person full and partial days out of role (DOR) associated with the mental and physical disorders in the Part II Saudi National Mental Health Survey (n = 1981)a. [file 12889_2022_12721_MOESM1_ESM.docx]

| **Supplementary Table 1. Incremental increases in total days out of role (DOR) associated with each of the mental and physical disorders in the Part II Saudi National Mental Health Survey (n=1,981)^a^** | | | | | | | |
| --- | --- | --- | --- | --- | --- | --- | --- |
|  | **Multivariable with RF prediction model^b^** | | |  | **Reduced multivariable model^c^** | | |
|  | **Coefficient** | **(95% CI)** | **χ^2^** |  | **Coefficient** | **(95% CI)** | **χ^2^** |
| **12-month mental disorders** |  |  |  |  |  |  |  |
| Anxiety disorder |  |  |  |  |  |  |  |
| Panic disorder or Agoraphobia | 0.83 | (-1.25-2.92) | 0.6 |  | 0.90 | (-1.16-2.97) | 0.7 |
| Separation anxiety or Social phobia | 1.35 | (-0.01-2.71) | 3.9 |  | 1.28 | (-0.05-2.61) | 3.6 |
| Generalized anxiety disorder | 1.67 | (-1.80-5.15) | 0.9 |  | - | - | - |
| Post-traumatic stress disorder | 1.09 | (-0.93-3.11) | 1.1 |  | 1.02 | (-0.76-2.80) | 1.3 |
| Obsessive-compulsive disorder | 1.02 | (-1.58-3.62) | 0.6 |  | 1.04 | (-1.43-3.51) | 0.7 |
| Mood disorder |  |  |  |  |  |  |  |
| Major depressive disorder | 2.94 | (0.39-5.49) | 5.2* |  | 2.78 | (0.54-5.03) | 6.0* |
| Bipolar I-II disorders | 0.59 | (-1.39-2.57) | 0.3 |  | - | - | - |
| Disruptive behavior disorder |  |  |  |  |  |  |  |
| ADHD | 2.13 | (0.28-3.99) | 5.2* |  | 2.05 | (0.19-3.91) | 4.8* |
| Intermittent explosive disorder | 2.03 | (-0.48-4.55) | 2.5 |  | 1.98 | (-0.56-4.52) | 2.4 |
| Any eating disorder | 1.34 | (-1.72-4.39) | 0.7 |  | 1.38 | (-1.45-4.20) | 0.9 |
| **12-month physical disorders** |  |  |  |  |  |  |  |
| Arthritis | 0.51 | (-0.44-1.46) | 1.1 |  | 0.47 | (-0.29-1.23) | 1.5 |
| Back/neck pain | 0.23 | (-0.57-1.02) | 0.3 |  | - | - | - |
| Headaches | -0.07 | (-0.58-0.44) | 0.1 |  | - | - | - |
| Other chronic pain | 0.77 | (-0.02-1.56) | 3.7 |  | 0.79 | (0.15-1.42) | 6.0* |
| Hypertension | -0.01 | (-0.70-0.67) | 0.0 |  | - | - | - |
| Heart disease | 0.93 | (-1.00-2.86) | 0.9 |  | - | - | - |
| Asthma | 0.27 | (-1.40-1.94) | 0.1 |  | - | - | - |
| Other lung disease | 26.99 | (21.84-32.13) | 107.4* |  | 27.65 | (24.35-30.96) | 273.2* |
| Injuries requiring hospitalization | 1.24 | (-0.57-3.04) | 1.8 |  | 1.23 | (-0.60-3.06) | 1.8 |
| Cancer | 0.28 | (-1.34-1.89) | 0.1 |  | - | - | - |
| Random forest predicted value (mean) | -0.06 | (-044-0.32) | 0.1 |  | - | - | - |
|  |  |  |  |  |  |  |  |

Abbreviations. RF, random forest; CI, confidence interval; ADHD, attention-deficit/hyperactivity disorder.

^a^Estimates come from linear regression models based on weighted Part II sample data. Total DOR are defined as the sum of (1) full days and (2) partial days divided by 2. All models included controls for age, gender, employment, education, and marital status.

^b^Multivariable model with a single continuous predicted number of days based on the random forest algorithm (χ^2^=0.1, p=.88).

^c^Reduced model based on LASSO regression results.

| **Supplementary Table 2. Incremental increases in full days out of role (DOR) associated with each of the mental and physical disorders in the Part II Saudi National Mental Health Survey (n=1,981)^a^** | | | | | | | | | | | |
| --- | --- | --- | --- | --- | --- | --- | --- | --- | --- | --- | --- |
|  | **Full days out of role** | | | | | | | | | | |
|  | **Univariable models** | | |  | **Multivariable model** | | |  | **Reduced multivariable model^b^** | | |
|  | **Coefficient** | **(95% CI)** | **χ^2^** |  | **Coefficient** | **(95% CI)** | **χ^2^** |  | **Coefficient** | **(95% CI)** | **χ^2^** |
| 12-month mental disorders |  |  |  |  |  |  |  |  |  |  |  |
| Anxiety disorder |  |  |  |  |  |  |  |  |  |  |  |
| Panic disorder or Agoraphobia | 1.77 | (-0.26-3.80) | 3.0 |  | 0.94 | (-1.03-2.91) | 0.9 |  | 0.99 | (-0.98-2.95) | 1.0 |
| Separation anxiety or Social phobia | 1.36 | (0.53-2.18) | 10.6* |  | 0.66 | (-0.37-1.69) | 1.6 |  | 0.71 | (-0.29-1.72) | 2.0 |
| Generalized anxiety disorder | 1.51 | (-0.61-3.63) | 2.0 |  | 0.59 | (-1.72-2.90) | 0.3 |  | - | - | - |
| Post-traumatic stress disorder | 1.06 | (-0.35-2.47) | 2.2 |  | 0.27 | (-1.09-1.63) | 0.2 |  | - | - | - |
| Obsessive-compulsive disorder | 0.87 | (-0.36-2.11) | 1.9 |  | -0.09 | (-1.56-1.39) | 0.0 |  | - | - | - |
| Mood disorder |  |  |  |  |  |  |  |  |  |  |  |
| Major depressive disorder | 2.78 | (0.51-5.04) | 5.9* |  | 2.11 | (-0.18-4.40) | 3.3 |  | 2.10 | (-0.13-4.32) | 3.5 |
| Bipolar I-II disorders | 1.89 | (0.24-3.54) | 5.1* |  | 0.55 | (-1.10-2.19) | 0.4 |  | - | - | - |
| Disruptive behavior disorder |  |  |  |  |  |  |  |  |  |  |  |
| ADHD | 2.03 | (0.73-3.33) | 9.5* |  | 1.10 | (-0.17-2.37) | 2.9 |  | 1.19 | (-0.14-2.52) | 3.1 |
| Intermittent explosive disorder | 2.89 | (0.83-4.95) | 7.7* |  | 1.54 | (-0.64-3.71) | 1.9 |  | 1.60 | (-0.59-3.79) | 2.1 |
| Any eating disorder | 2.23 | (-0.66-5.12) | 2.3 |  | 1.34 | (-1.58-4.26) | 0.8 |  | 1.43 | (-1.40-4.26) | 1.0 |
| 12-month physical disorders |  |  |  |  |  |  |  |  |  |  |  |
| Arthritis | 1.91 | (-0.70-4.52) | 2.1 |  | 0.21 | (-0.44-0.86) | 0.4 |  | - | - | - |
| Back/neck pain | 0.16 | (-0.57-0.89) | 0.2 |  | -0.10 | (-0.63-0.43) | 0.1 |  | - | - | - |
| Headaches | 0.15 | (-0.32-0.62) | 0.4 |  | -0.08 | (-0.49-0.33) | 0.1 |  | - | - | - |
| Other chronic pain | 0.50 | (-0.23-1.23) | 1.8 |  | 0.39 | (-0.09-0.88) | 2.6 |  | - | - | - |
| Hypertension | -0.30 | (-1.40-0.80) | 0.3 |  | -0.04 | (-0.55-0.46) | 0.0 |  | - | - | - |
| Heart disease | 9.99 | (-2.84-22.83) | 2.4 |  | 0.09 | (-1.28-1.47) | 0.0 |  | - | - | - |
| Asthma | 0.47 | (-0.73-1.67) | 0.6 |  | -0.04 | (-1.12-1.05) | 0.0 |  | - | - | - |
| Other lung disease | 28.34 | (25.38-31.30) | 358.1* |  | 27.74 | (24.02-31.47) | 217.0* |  | 28.00 | (24.79-31.20) | 298.8* |
| Injuries requiring hospitalization | 1.12 | (-0.64-2.88) | 1.6 |  | 1.08 | (-0.76-2.92) | 1.3 |  | 1.10 | (-0.64-2.84) | 1.6 |
| Cancer | -0.43 | (-1.20-0.33) | 1.2 |  | -0.16 | (-0.78-0.47) | 0.3 |  | - | - | - |
|  |  |  |  |  |  |  |  |  |  |  |  |

Abbreviations. CI, confidence interval; ADHD, attention-deficit/hyperactivity disorder.

^a^Estimates come from linear regression models based on weighted Part II sample data. All models included controls for age, gender, employment, education, and marital status. The 20 univariable models added only one mental or physical disorder as a predictor to these control variables. The multivariable model added all 20 disorders to the control variables.

^b^Reduced model based on LASSO regression results.

| **Supplementary Table 3. Incremental increases in partial days out of role (DOR) associated with each of the mental and physical disorders in the Part II Saudi National Mental Health Survey (n=1,981)^a^** | | | | | | | | | | | |
| --- | --- | --- | --- | --- | --- | --- | --- | --- | --- | --- | --- |
|  | **Partial days out of role** | | | | | | | | | | |
|  | **Univariable models** | | |  | **Multivariable model** | | |  | **Reduced multivariable model^b^** | | |
|  | **Coefficient** | **(95% CI)** | **χ^2^** |  | **Coefficient** | **(95% CI)** | **χ^2^** |  | **Coefficient** | **(95% CI)** | **χ^2^** |
| 12-month mental disorders |  |  |  |  |  |  |  |  |  |  |  |
| Anxiety disorder |  |  |  |  |  |  |  |  |  |  |  |
| Panic disorder or Agoraphobia | 0.81 | (0.11-1.52) | 5.2* |  | -0.17 | (-0.94-0.60) | 0.2 |  | - | - | - |
| Separation anxiety or Social phobia | 1.71 | (0.77-2.65) | 12.9* |  | 1.02 | (0.12-1.92) | 5.0* |  | 1.13 | (0.25-2.01) | 6.4* |
| Generalized anxiety disorder | 2.14 | (-0.58-4.86) | 2.4 |  | 1.50 | (-1.54-4.55) | 0.9 |  | - | - | - |
| Post-traumatic stress disorder | 2.36 | (0.68-4.03) | 7.7* |  | 1.40 | (-0.51-3.31) | 2.1 |  | 1.37 | (-0.49-3.23) | 2.1 |
| Obsessive-compulsive disorder | 2.95 | (0.56-5.33) | 6.0* |  | 2.14 | (-0.30-4.59) | 3.0 |  | 2.22 | (-0.12-4.56) | 3.5 |
| Mood disorder |  |  |  |  |  |  |  |  |  |  |  |
| Major depressive disorder | 1.79 | (0.65-2.94) | 9.5* |  | 1.36 | (0.26-2.46) | 6.0* |  | 1.45 | (0.40-2.51) | 7.4* |
| Bipolar I-II disorders | 1.32 | (-0.06-2.70) | 3.6 |  | -0.02 | (-1.43-1.39) | 0.0 |  | - | - | - |
| Disruptive behavior disorder |  |  |  |  |  |  |  |  |  |  |  |
| ADHD | 2.55 | (0.95-4.15) | 9.9* |  | 1.78 | (0.41-3.16) | 6.5* |  | 1.87 | (0.31-3.43) | 5.6* |
| Intermittent explosive disorder | 2.11 | (0.57-3.64) | 7.4* |  | 0.88 | (-0.68-2.44) | 1.2 |  | - | - | - |
| Any eating disorder | 0.59 | (-0.21-1.39) | 2.1 |  | -0.14 | (-0.93-0.66) | 0.1 |  | - | - | - |
| 12-month physical disorders |  |  |  |  |  |  |  |  |  |  |  |
| Arthritis | 0.81 | (0.12-1.49) | 5.4* |  | 0.33 | (-0.33-1.00) | 1.0 |  | 0.29 | (-0.32-0.91) | 0.9 |
| Back/neck pain | 1.07 | (0.42-1.71) | 10.6* |  | 0.50 | (-0.23-1.22) | 1.8 |  | 0.60 | (-0.09-1.29) | 3.0 |
| Headaches | 0.56 | (0.12-1.00) | 6.3* |  | 0.11 | (-0.29-0.50) | 0.3 |  | - | - | - |
| Other chronic pain | 1.05 | (0.35-1.75) | 8.9* |  | 0.72 | (0.01-1.42) | 4.0* |  | 0.76 | (0.05-1.47) | 4.5* |
| Hypertension | 0.35 | (-0.41-1.11) | 0.8 |  | -0.01 | (-0.74-0.73) | 0.0 |  | 0.07 | (-0.68-0.83) | 0.0 |
| Heart disease | 1.30 | (-0.49-3.09) | 2.1 |  | 1.41 | (-0.68-3.50) | 1.8 |  | 0.89 | (-0.67-2.45) | 1.3 |
| Asthma | 1.07 | (0.02-2.12) | 4.1* |  | 0.53 | (-0.61-1.68) | 0.8 |  | 0.56 | (-0.57-1.69) | 1.0 |
| Other lung disease | 0.08 | (-0.71-0.88) | 0.0 |  | -1.51 | (-3.75-0.73) | 1.8 |  | - | - | - |
| Injuries requiring hospitalization | 0.58 | (0.10-1.06) | 5.7* |  | 0.22 | (-0.28-0.73) | 0.8 |  | - | - | - |
| Cancer | 0.57 | (-3.04-4.18) | 0.1 |  | 0.93 | (-2.17-4.03) | 0.4 |  | 1.00 | (-2.13-4.13) | 0.4 |
|  |  |  |  |  |  |  |  |  |  |  |  |

Abbreviations. CI, confidence interval; ADHD, attention-deficit/hyperactivity disorder.

^a^Estimates come from linear regression models based on weighted Part II sample data. All models included controls for age, gender, employment, education, and marital status. The 20 univariable models added only one mental or physical disorder as a predictor to these control variables. The multivariable model added all 20 disorders to the control variables.

^b^Reduced model based on LASSO regression results.

| **Supplementary Table 4. Population attributable risk percent (PARP) and incremental annualized per person full and partial days out of role (DOR) associated with the mental and physical disorders in the Part II Saudi National Mental Health Survey (n=1,981)^a^** | | | | | | | | |
| --- | --- | --- | --- | --- | --- | --- | --- | --- |
|  | **12-month mental disorders** | |  | **12-month physical disorders** | |  | **Neither** | |
|  | **Estimate** | **(SE)** |  | **Estimate** | **(SE)** |  | **Estimate** | **(SE)** |
| **I. Prevalence (%)** |  |  |  |  |  |  |  |  |
| Full days out of role | 19.9 | (1.3) |  | 47.1 | (2.2) |  | 45.5 | (2.5) |
| Partial days out of role | 19.9 | (1.3) |  | 47.1 | (2.2) |  | 45.5 | (2.5) |
| **II. PARP (%)^b^** |  |  |  |  |  |  |  |  |
| Full days out of role | 32.1 | (8.9) |  | 26.1 | (11.6) |  | 41.9 | (6.9) |
| Partial days out of role | 34.9 | (4.6) |  | 28.8 | (6.7) |  | 36.3 | (6.8) |
| **III. Inc**re**mental annualized per person total disability days (Total sample)^c^** | | | | | | | | |
| Full days out of role | 3.6 | (1.0) |  | 3.0 | (1.3) |  | - | - |
| Partial days out of role | 3.4 | (0.4) |  | 2.8 | (0.6) |  | - | - |
| **IV. Incremental annualized per person total disability days (Among people with the disorders)^d^** | | | | | | | | |
| Full days out of role | 18.3 | (5.1) |  | 6.3 | (2.8) |  | 4.8 | (0.8) |
| Partial days out of role | 17.0 | (2.2) |  | 5.9 | (1.4) |  | 3.5 | (0.7) |
|  |  |  |  |  |  |  |  |  |

Abbreviations. SE, standard error.

^a^Estimates are based on weighted Part II sample data.

^b^PARP estimates are generated from the multivariable model in Table 4.

^c^The estimates of annualized days are based on PARP estimates multiplied by the mean full or partial DOR in the last 30 days times 12. The mean full days out of role in the Part II sample equals 0.9 and mean partial days out of role equals 0.8.

^d^Sub-group annualized days equal total annualized days multiplied by 1/(prevalence of the subgroup in the total sample).
